# Risks of malignancies related to disease-modifying antirheumatic drugs in rheumatoid arthritis: a pharmacovigilance analysis using the FAERS database

**DOI:** 10.3389/fphar.2024.1458500

**Published:** 2024-11-13

**Authors:** Wan Xiong, Yilin Li, Lin Hu, Gefei He, Juanjuan Huang

**Affiliations:** ^1^ Department of Pharmacy, The Affiliated Changsha Hospital of Xiangya School of Medicine, Central South University, Changsha, China; ^2^ Department of Pharmacy, The First Hospital of Changsha, Changsha, China; ^3^ Department of Information and Digital Technology, PowerChina Zhongnan Engineering Corporation Limited, Changsha, China

**Keywords:** DMARDs, malignancy, rheumatoid arthritis, FAERS, pharmacovigilance

## Abstract

**Objectives:**

Over the years when disease-modifying antirheumatic drugs (DMARDs) have been used in rheumatoid arthritis patients, reports of malignancies have emerged. This study aims to investigate the association between malignancies and DMARDs by using data extracted from the Food and Drug Administration Adverse Event Reporting System (FAERS).

**Methods:**

FAERS data (January 2019 to December 2023) were reviewed. For each drug-event pair, the disproportionality analysis was conducted to evaluate the risk of malignancy. Multivariate logistic regression was implemented to mitigate potential biases. Moreover, the time to onset of malignancy was also evaluated.

**Results:**

We conducted a detailed search for rheumatoid arthritis indications and identified a total of 17,412 adverse event reports associated with malignancies, with selective DMARDs designated as the role code “primary suspect”. At the preferred term level, there were 198 positive signals, among which the lower limit of the 95% confidence interval for the information component is 3.55 for squamous cell carcinoma of the skin, 2.39 for breast cancer, and 2.27 for lymphoproliferative disorder. In comparison to other DMARDs, targeted synthetic DMARDs were associated with a broader range of malignancies at both preferred term and Standardized MedDRA Queries levels. The number of adverse events reported in female patients is approximately 2–3 times higher than men, and the median age across the population was approximately 62 years. In terms of onset time, the conventional synthetic DMRADs exhibited a relatively longer median time, ranging from 3.58 to 7.08 years, while the targeted synthetic DMARDs demonstrated a shorter median time of 0.83–1.67 years.

**Conclusion:**

Our study uncovers varying degrees of malignancy risks related to DMARDs, with a significantly higher risk observed in targeted synthetic DMARDs. Additionally, novel malignancy signals, not documented in product labels, have been detected. In the future, further research will be necessary to validate our findings.

## 1 Introduction

Rheumatoid arthritis (RA) is a systemic autoimmune disorder characterized by the immune system’s assault on the synovial tissues of joints across the body. This condition is marked by a relentless progression of joint damage and extra-articular symptoms. Without intervention, disease progression can lead to various clinical symptoms, potentially culminating in irreversible disabilities (Aletaha and Smolen, 2018). Being neither preventable nor curable, RA significantly diminishes the quality of life and imposes a substantial societal burden. The 2017 Global Burden of Disease Study highlights a worldwide prevalence of RA at 0.27% (Vos et al., 2020). A recent Chinese survey revealed that the annual average direct cost for each RA patient amounts to $1,917.21 ± $2,559.06 (Hu et al., 2018). Beyond direct healthcare expenses, the functional impairment and diminished work capacity among RA patients, coupled with reduced social engagement, contribute to a significant socio-economic strain (Sokka et al., 2010).

RA is also associated with a heightened prevalence of comorbidities, including an increased risk of certain malignancies such as lymphoma and lung cancer (De Cock and Hyrich, 2018). The association between RA and malignancy was first delineated in Isomäki et al. (1978), who observed that individuals with RA faced a higher likelihood of developing lymphoma. This seminal finding spurred a wave of research into this potential connection (Pundole and Suarez-Almazor, 2020). The etiology of RA’s increased malignancy risk are likely multifaceted, encompassing chronic inflammation and certain risk factors shared with malignancy (Singh and Li, 2021). Furthermore, the escalating use of DMARDs in the management of RA has ignited concerns regarding whether these treatments might inadvertently heighten the risk of certain malignancies.

DMARDs are a class of drugs that can interfere with the signs and symptoms of RA and inhibit the progression of structural joint damage. Current approved DMARDs in RA include conventional synthetic DMARDs (csDMARDs): methotrexate, leflunomide, sulfasalazine, hydroxychloroquine; targeted synthetic DMARDs (tsDMARDs): pan-Janus kinase- and Janus kinase1/2-inhibitors (JAKi) such as upadacitinib, baricitinib, tofacitinib, filgotinib and peficitinib; biologic DMARDs (bDMARDs): tumor necrosis factor-α (TNF-α) inhibitor (etanercept, infliximab, adalimumab, certolizumab pegol, and golimumab), interleukin 6 inhibitors (tocilizumab and sarilumab), interleukin-1 inhibitors (anakinra), B cell depleting antibodies (rituximab), and inhibitors of co-stimulatory molecules (abatacept) (Sepriano et al., 2023; Ding et al., 2023). In light of the 2021 preliminary safety trial outcomes, the Food and Drug Administration (FDA) disseminated documentation and advisories, signaling that tofacitinib could potentially increase the risk of malignancy when compared to TNF-α inhibitors (TNFi) (FDA, 2022a). The Huss cohort study indicated an elevated risk of non-melanoma skin cancer in patients using JAKi (Huss et al., 2023). Bongartz reported a dose-dependent escalation in malignancy risk for RA patients treated with TNFi (Bongartz et al., 2006). Additionally, a German study by Strangfeld noted a possible uptick in pancreatic cancer incidence linked to leflunomide use (Strangfeld et al., 2010). Collectively, these findings raise concerns that DMARDs might carry an inherent, irreversible risk of malignancies. However, there is currently limited evidence regarding the malignancy risks of DMARDs, and comparative studies on the tumor risks among different DMARDs are also limited. The relationship between DMARDs and malignancy risks in real-world scenarios cannot be conclusively elucidated.

FAERS database serves as a global spontaneous reporting system, amassing a vast trove of real-world data on adverse events (AEs). It has been widely used to identify AE risk signals. The aim of this study was to use standardized data from FAERS to assess malignancy risks associated with various DMARDs.

## 2 Methods

### 2.1 Data sources

We conducted a pharmacovigilance study on malignancy risk associated with DMARDs based on the FAERS database, a publicly available database of safety reports submitted by healthcare professionals, patients, drug manufacturers, and more (Zhou et al., 2023). It encompasses seven types of data documents, including DEMO (demographic and administrative information), DRUG (drug/biologic information), INDI (indications for drug administration), REAC (coded adverse events), OUTC (patient outcomes), RPSR (report sources), and THER (therapy start and end dates for reported drugs) (Chen et al., 2023).

AEs in the FAERS database are reported using the preferred term (PT) codes from the Medical Dictionary for Regulatory Activities (MedDRA), which is logically organized into five hierarchical levels. PT serve as unique descriptors for a single medical concept, encompassing symptoms, signs, diseases, diagnoses, indications, examination, etc. Additionally, PT can be categorized into high-level terms (HLT), high-level group terms (HLGT) and system organ class (SOC), or clustered using Standardized MedDRA Queries (SMQs) for specific medical conditions (Lin et al., 2020).

### 2.2 Procedures

**FIGURE 1:**
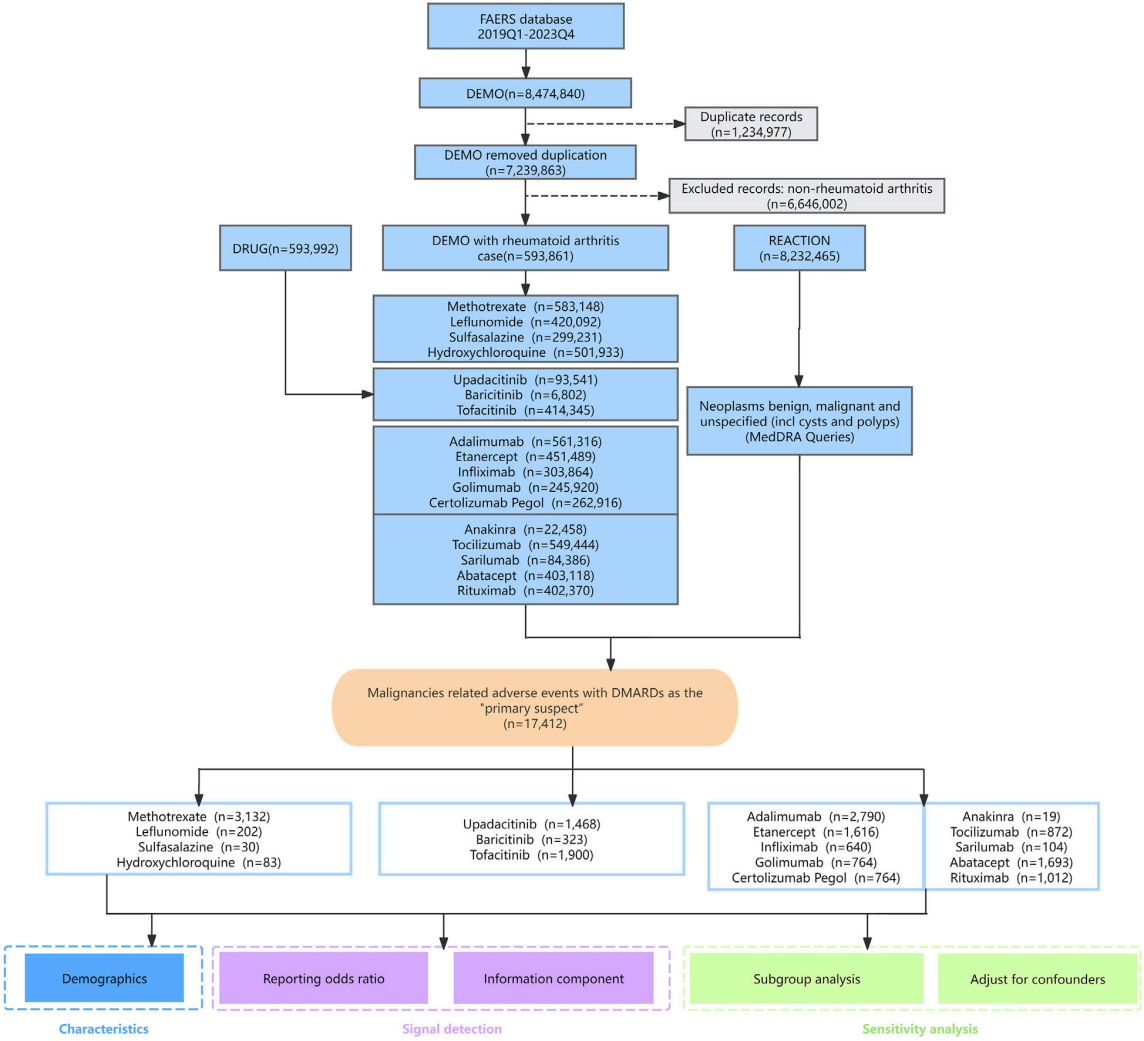
The flow chart of the study.

A total of 8,474,840 raw reports from first quarter (Q1) of 2019 to Q4 2023 in FAERS were imported into a MySQL database using python pandas (version 2.1.0) for data cleaning and connection. To eliminate duplicate and multiple reports, the variable matching method, as recommended by the FDA, was employed (Tregunno et al., 2014). Delete duplicate cases according to the delete file package and selects the latest FDA_DT and the higher PRIMARYID when CASEID is the same (Chen et al., 2024). This process resulted in the removal of 1,234,977 duplicated reports (Figure 1).

To rule out the influence of underlying diseases, we limited our study population to patients with RA, and the analysis set ultimately included 593,861 reports. Twenty types of DMARDs, including csDMARDs (methotrexate, leflunomide, sulfasalazine, hydroxychloroquine), bDMARDs (adalimumab, etanercept, infliximab, golimumab, certolizumab pegol, anakinra, tocilizumab, sarilumab, abatacept, rituximab), and tsDMARDs (iguratimod, upadacitinib, baricitinib, tofacitinib, filgotinib, peficitinib) (Chen et al., 2024; Siebert et al., 2015), were used as keywords to obtain report data from the FAERS Publish Dashboard, and only cases with a reported role code of “primary suspect” (PS) were included to improve accuracy and obtain better signal intensity. “Neoplasms benign, malignant and unspecified (incl cysts and polyps)” (Code: 10029104) were identified according to the Medical Dictionary for Regulatory Activities (MedDRA) terminology to discribe the cancer risk related to DMARDs. Specifically, we also grouped AEs into SMQ to describe disease conditions and eventually focused on 9 SMQs associated with cancer risk incorporating malignancies; breast neoplasms, malignant and unspecified; skin neoplasms, malignant and unspecified; premalignant disorders; malignant lymphomas; prostate neoplasms, malignant and unspecified; ovarian neoplasms, malignant and unspecified; uterine and fallopian tube neoplasms, malignant and unspecified and tumour lysis syndrome.

### 2.3 Statistical analysis

To assess a potential pattern of AEs, information regarding patient demographics, including gender, age, reporting countries and outcomes were analyzed. Meanwhile, time to onset which defined as the duration between the initiation of DMARDs treatment and the occurrence of malignancies was examined separately. Categorical variables were summarized using percentages and frequencies, while continuous variables were expressed in medians with interquartile ranges.

In pharmacovigilance analysis, disproportionality analysis (DPA) has been used as a data mining algorithm to identify drug-AE signals in spontaneous reporting systems by calculating the signal score (Tyagi and Kumar, 2024). In this study, DPA was evaluated using the reporting odds ratio (ROR) and the information component (IC) to detect an increased reporting of DMARDs-associated malignancy AEs compared to other reports within RA during the same period. A signal was confirmed when, in at least 3 reports, the lower 95% confidence interval for ROR (ROR_025_) exceeded 1 and that for information components (IC_025_) was greater than 0 (Jain et al., 2023). The equations of ROR and IC are listed in Supplementary Table S1.

In the treatment regimen of RA, a common approach to enhance therapeutic efficacy involves combining DMARDs with additional medications. Given the likelihood of multiple concurrent health issues in patients, polypharmacy frequently occurs. This practice of using multiple medications concurrently can influence the onset and progression of malignancy risks. To bolster the robustness of our results and mitigate potential biases, we utilized multivariate logistic regression to control for factors such as age, gender, and the use of concomitant medications. Through a comprehensive statistical analysis of co-administration, we incorporated medications that might affect tumor-related outcomes into our regression model.

## 3 Results

### 3.1 Clinical characteristics

A total of 8,474,840 AE reports were involved in the FAERS database from Q1 2019 to Q4 2023. After the exclusion of duplicates, 17,412 reports of malignancy AEs with DMARDs as the “primary suspect” were identified. Through the analysis of the distribution of spontaneous reports for each DMARDs, csDMARDs were implicated in 3,447 (19.80%) cases, bDMARDs in 10,274 (59.01%) and tsDMARDs in 3,691 (21.20%). Methotrexate was the most frequently reported with 3,132 (17.99%) reports, followed by adalimumab with 2,790 (16.02%), and tofacitinib with 1,900 (10.91%). Abatacept was mentioned in 1,693 (9.72%) reports, and etanercept in 1,616 (9.28%). Of note, there were no relevant reports related to iguratimod, filgotinib and peficitinib.

The clinical characteristics of these patients are described in Supplementary Table S2. The number of AEs reported in female patients was approximately 2–3 times higher than in male patients. The median age across the population was approximately 62 years. Specifically, adalimumab and sulfasalazine had median ages of 58.8 and 73 years, respectively. The largest proportion of reports came from the United States and Canada, with 4,482 (26.31%) and 3,914 (22.97%) reports, respectively. Regarding the outcomes of AEs, other important medical events were identified in 14,413 (62.14%) reports. Additionally, hospitalization was the most frequent outcome among these cases, occurring in 5,249 (22.63%) instances. Death and risk to life were reported in 1,618 (6.98%) and 1,232 (5.31%) cases, respectively, while disability was noted in 631 (2.72%) cases.

### 3.2 The spectrum of malignancy risks at the PT level

A total of 198 positive signals at the PT level were identified. Methotrexate, upadacitinib and adalimumab were involved in more PT signals, with 40 (IC_025_ range: 0.01–2.59), 47 (IC_025_ range: 0.03–3.55) and 42 signals (IC_025_ range: 0.03–1.54), respectively (Supplementary Table S3).

**FIGURE 2:**
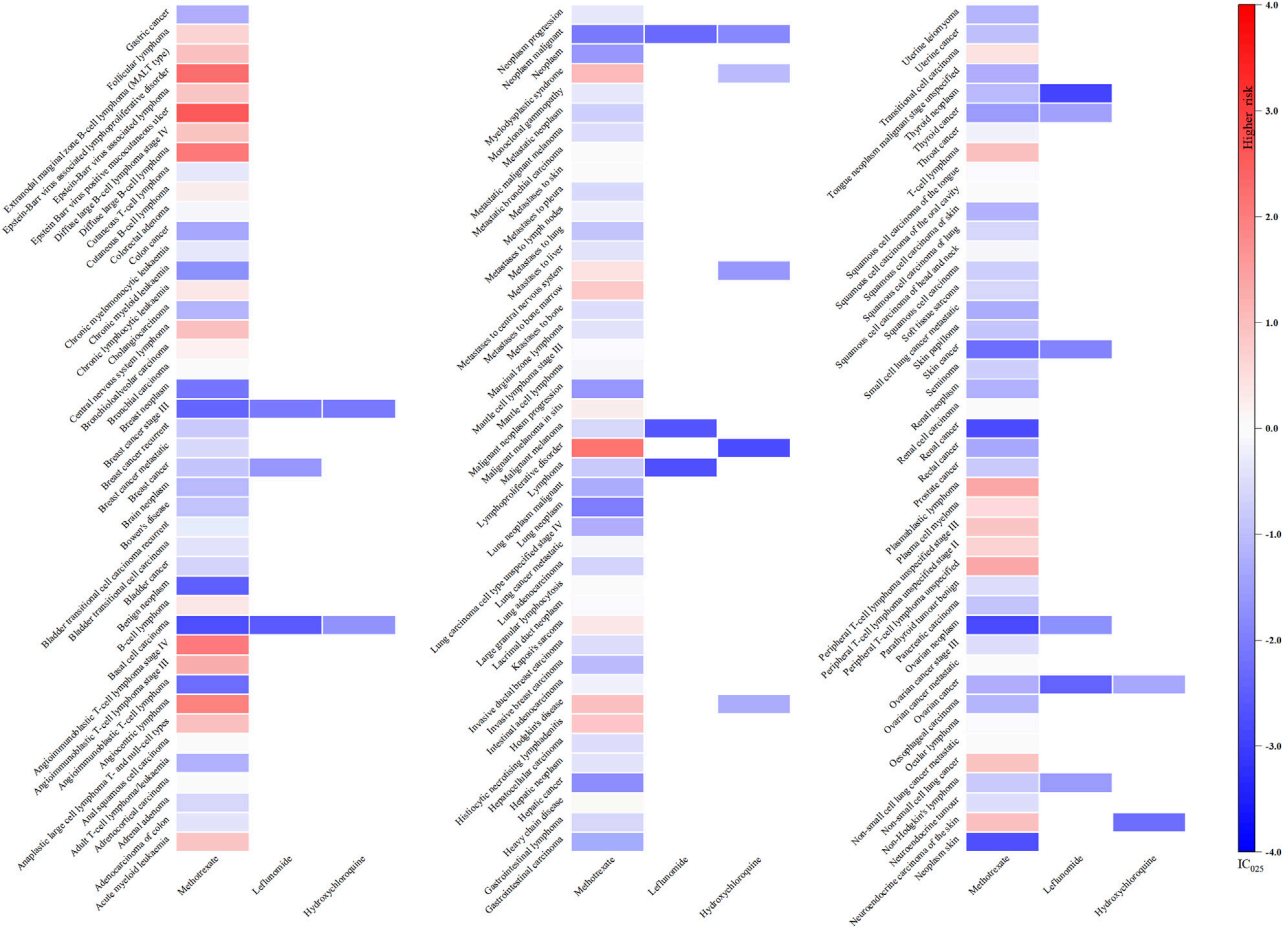
Heatmap showing the associations between csDMARDs and malignancy adverse events. Note: Sulfasalazine has not been reported as “primary suspect” drug and is therefore not included in the figure.

As illustrated in Figure 2, only methotrexate had 40 positive signals in the csDMARDs, including Epstein-Barr virus (EBV) positive mucocutaneous ulcer (IC_025_: 2.59), EBV associated lymphoproliferative disorder (IC_025_: 2.27), lymphoproliferative disorder (IC_025_: 2.20), diffuse large B-cell lymphoma (IC_025_: 2.07), and angiocentric lymphoma (IC_025_: 1.95).

**FIGURE 3:**
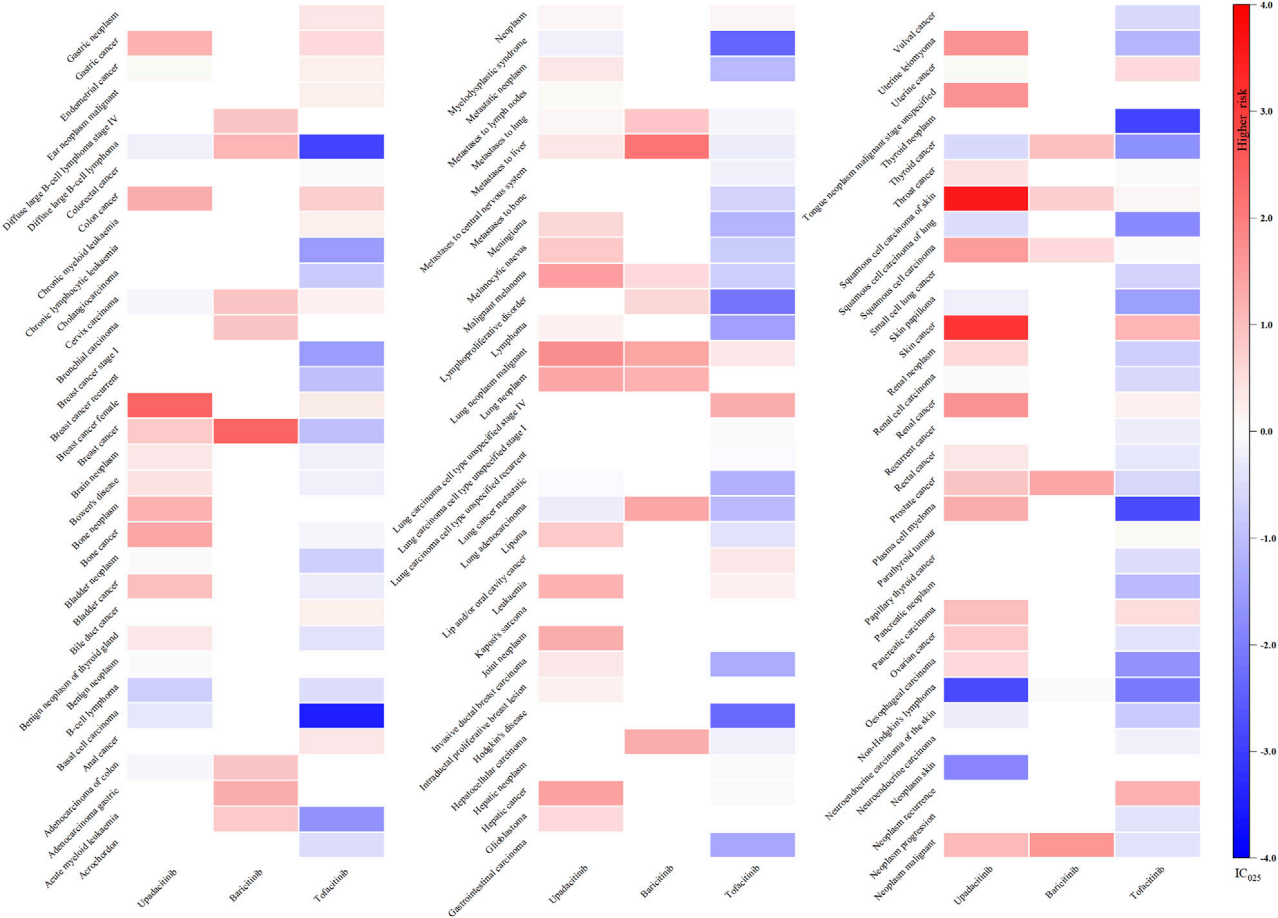
Heatmap showing the associations between tsDMARDs and malignancy adverse events. Note: Iguratimod, filgotinib, and peficitinib lack relevant reports and are therefore not included in the figure.

For tsDMARDs, upadacitinib, with 47 signals, exhibited a significant correlation with squamous cell carcinoma of skin (IC_025_: 3.55), skin cancer (IC_025_: 3.13), breast cancer female (IC_025_: 2.44), lung neoplasm malignant (IC_025_: 1.73), uterine leiomyoma (IC_025_: 1.68), renal cancer (IC_025_: 1.67), tongue neoplasm malignant stage unspecified (IC_025_: 1.66) and squamous cell carcinoma (IC_025_: 1.52). Specifically, baricitinib, with 22 positive signals, demonstrated the most potent association with breast cancer (IC_025_: 2.39), metastases to liver (IC_025_: 2.14) and neoplasm malignant (IC_025_: 1.65). Additionally, tofacitinib had 24 positive signals and the lung carcinoma cell type unspecified stage IV (IC_025_: 1.22), neoplasm recurrence (IC_025_: 1.20) and skin cancer (IC_025_: 1.13) were relatively noteworthy (Figure 3).

**FIGURE 4:**
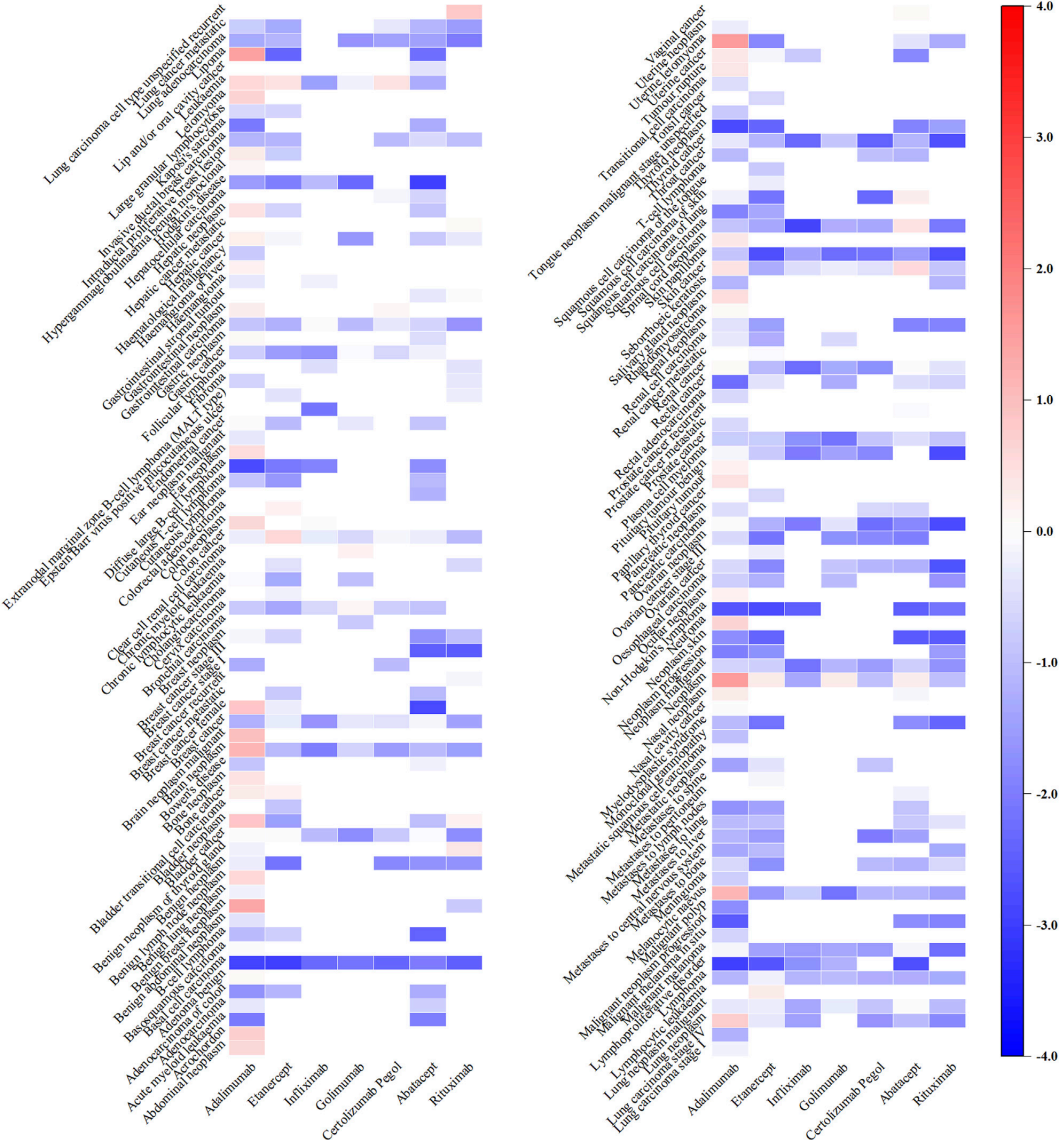
Heatmap showing the associations between bDMARDs and malignancy adverse events. Note: Anakinra, tocilizumab, and sarilumab lack significant signals and are therefore not included in the figure.

Regarding bDMARDs, a total of 65 positive signals were identified. More than 64% of these signals were related to adalimumab, with uterine leiomyoma (IC_025_: 1.54), neoplasm (IC_025_: 1.51), lipoma (IC_025_: 1.46), benign breast neoplasm (IC_025_: 1.37), melanocytic naevus (IC_025_: 1.13) and brain neoplasm (IC_025_: 1.12) exhibiting particularly strong positive signals. Additionally, rituximab showed signals related to lung carcinoma cell type unspecified recurrent (IC_025_: 0.84), benign neoplasm of thyroid gland (IC_025_: 0.38). Several other strong signals were also unveiled, including skin cancer (IC_025_: 0.56) and squamous cell carcinoma (IC_025_: 0.43) linked to abatacept, colon cancer (IC_025_: 0.57) and leukaemia (IC_025_: 0.45) linked to etanercept, lung carcinoma cell type unspecified recurrent (IC_025_: 0.84) associated with rituximab, and leukaemia (IC_025_: 0.45) related to certolizumab pegol (Figure 4).

### 3.3 Association signal detection at the SMQ level

**FIGURE 5:**
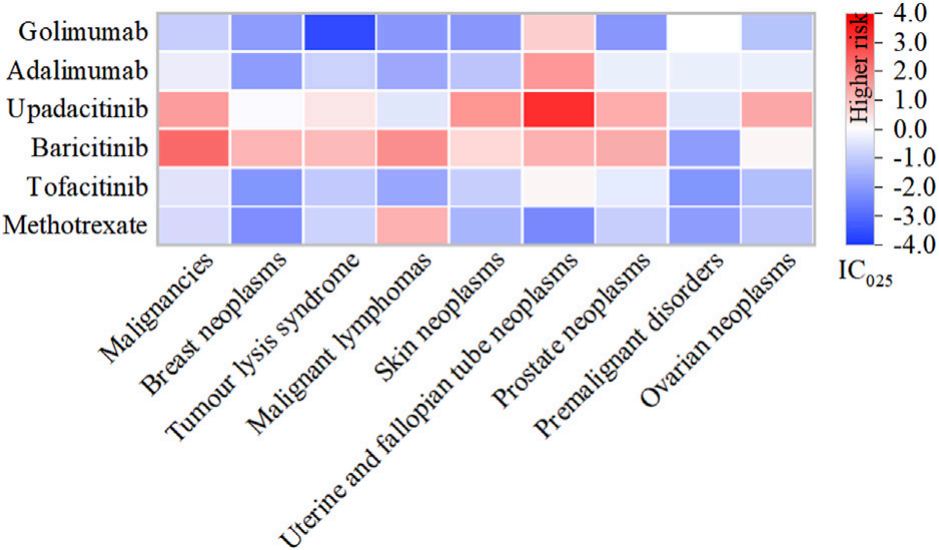
Heatmap under SMQs classification of different DMARDs. Note: DMARDs with a positive signal under the SMQ classification are displayed.

Among the 9 categories of SMQs, malignancies (N = 23,665, 59.07%) comprised the most frequently reported AEs related to tumour, followed by tumour lysis syndrome (N = 4,336, 10.82%), skin neoplasms, malignant and unspecified (N = 3,001, 7.49%) and breast neoplasms, malignant and unspecified (N = 2,998, 7.48%) (Figure 5).

Specifically, baricitinib had the most statistically significant AE signals, including malignancies (IC_025_: 2.31), malignant lymphomas (IC_025_: 1.74), prostate neoplasms, malignant and unspecified (IC_025_: 1.32), uterine and fallopian tube neoplasms, malignant and unspecified (IC_025_: 1.21), breast neoplasms, malignant and unspecified (IC_025_: 1.17), tumour lysis syndrome (IC_025_: 1.08), skin neoplasms, malignant and unspecified (IC_025_: 0.57) and ovarian neoplasms, malignant and unspecified (IC_025_: 0.14); Upadacitinib exhibited robust risk signals for uterine and fallopian tube neoplasms, malignant and unspecified (IC_025_: 3.26), skin neoplasms, malignant and unspecified (IC_025_: 1.64), malignancies (IC_025_: 1.53), ovarian neoplasms, malignant and unspecified (IC_025_: 1.34), prostate neoplasms, malignant and unspecified (IC_025_: 1.27); Both adalimumab (IC_025_: 1.61), golimumab (IC_025_: 0.72) along with tofacitinib (IC_025_: 0.12), were potentially associated with uterine and fallopian tube neoplasms, malignant and unspecified. Additionally, methotrexate displayed a connection with malignant lymphomas (IC_025_: 1.21) (Supplementary Table S4).

### 3.4 Sensitivity analysis

**FIGURE 6:**
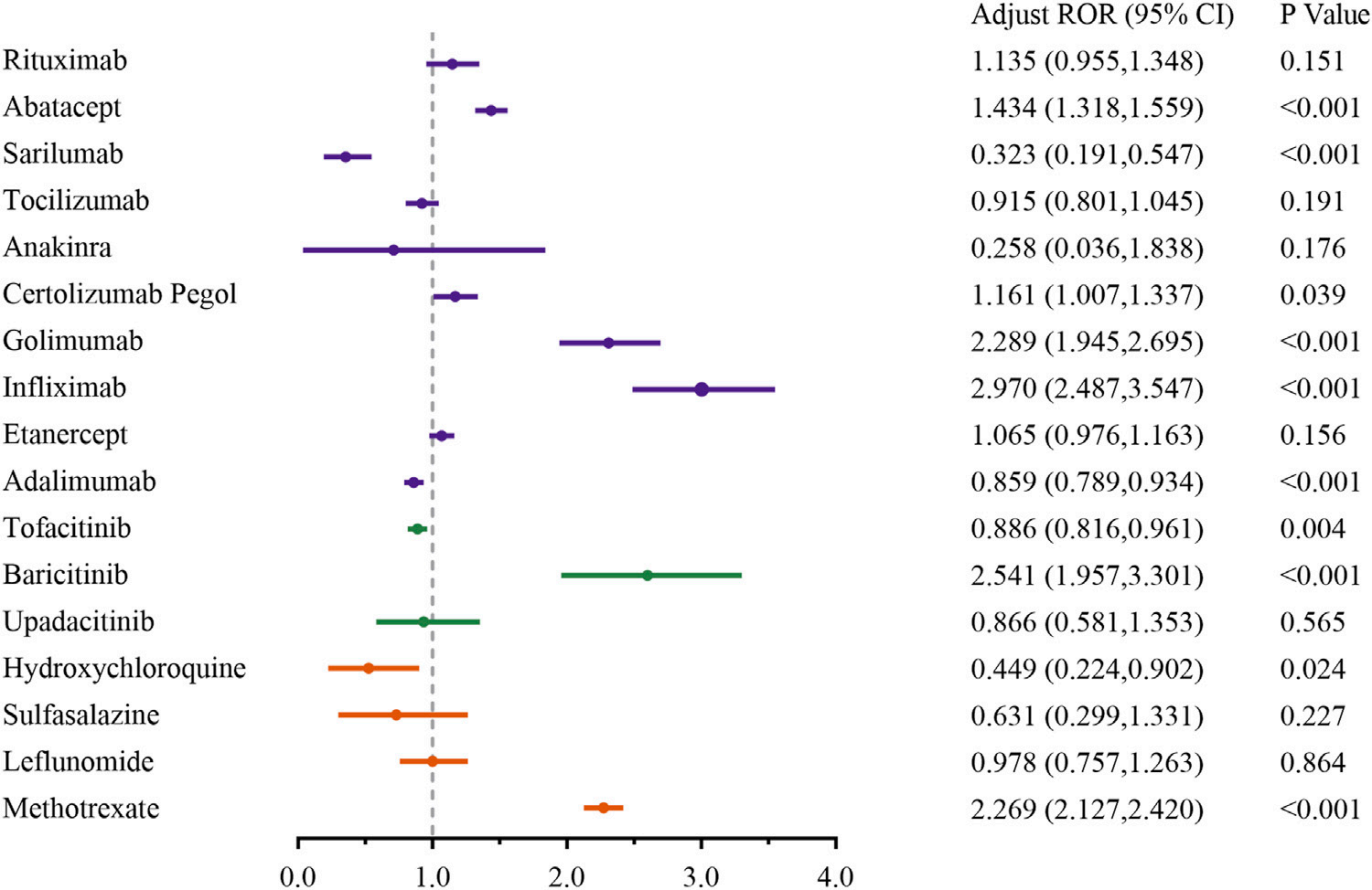
Sensitivity analysis of malignancy adverse events associated with DMARDs. Note: Adjust ROR, adjusted for age, gender and concomitant medications (methotrexate, tofacitinib citrate, leflunomide, and sulfasalazine) via a multivariable logistic regression. Orange represents csDMARDs, green represents tsDMARDs, and purple represents bDMARDs.

To minimize the impact of possible confounders, such as medication combinations, we detailed the malignancy risks of the top 10 concomitant drugs in Supplementary Table S5. Based on FDA label information, we noted that frequently co-administered medications, including methotrexate, tofacitinib, leflunomide and sulfasalazine, could potentially contribute to AEs related to malignancy. Therefore, these four drugs, along with age and gender, were included as confounding variables in a multivariate logistic regression model. Additionally, to mitigate the impact of diverse indications, the analysis was restricted to RA patients undergoing treatment with DMARDs. The findings, presented in Figure 6, clearly show a strong link between methotrexate (Adjust ROR: 2.269; 95%CI: 2.127–2.420), baricitinib (Adjust ROR: 2.541; 95%CI: 1.957,3.301), infliximab (Adjust ROR: 2.970; 95%CI: 2.487–3.547), golimumab (Adjust ROR: 2.289; 95%CI: 1.945–2.695), certolizumab Pegol (Adjust ROR: 1.161; 95%CI: 1.007–1.337), abatacept (Adjust ROR: 1.434; 95%CI: 1.318–1.559) and malignancy-related AEs, regardless of the adjustment for confounding factors.

### 3.5 Time to onset of malignancy adverse event

**FIGURE 7:**
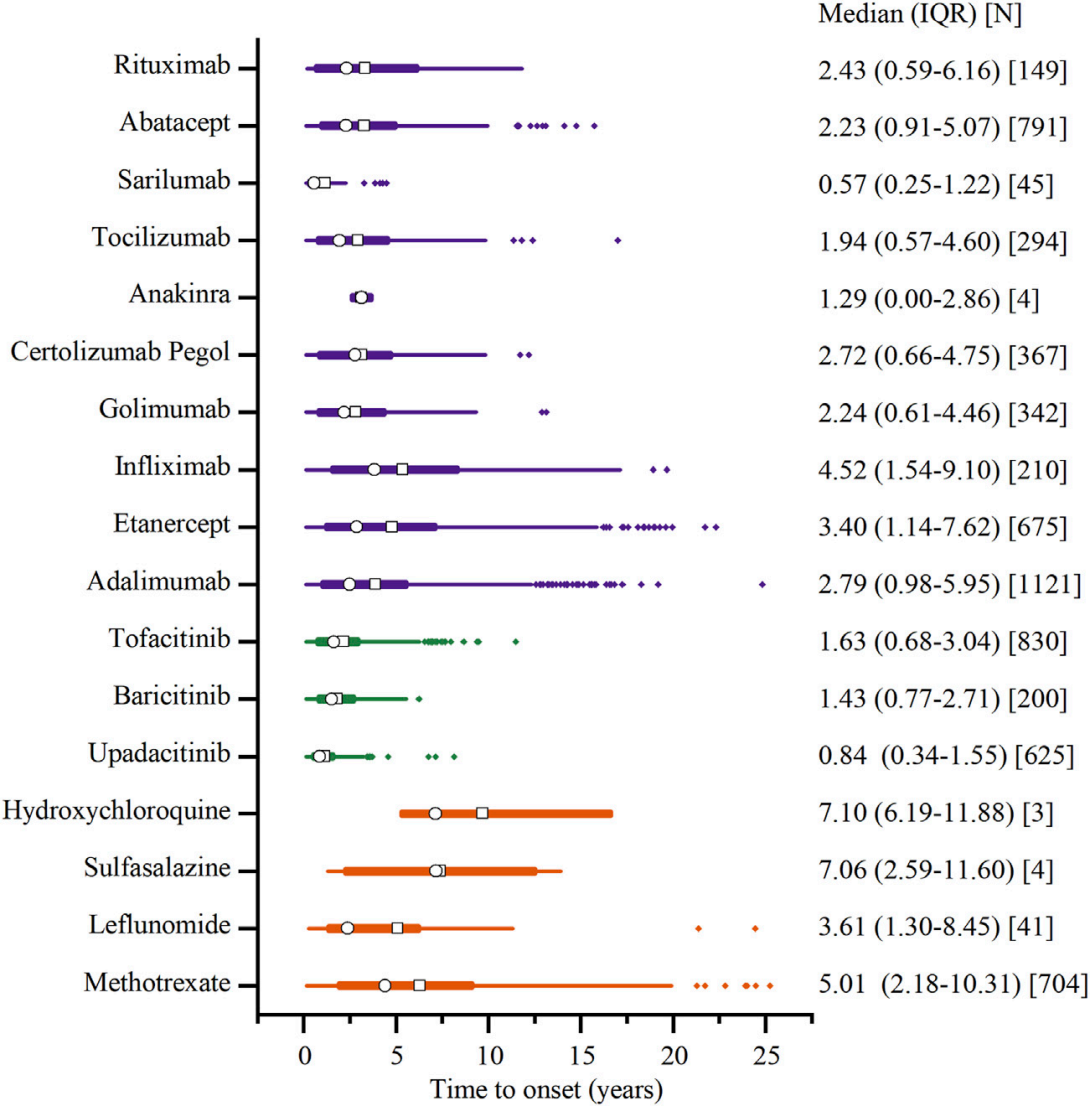
Time to onset of malignancy adverse events. Note: The □ represents the mean, and the ○ represents the median. Orange represents csDMARDs, green represents tsDMARDs, and purple represents bDMARDs.

Figure 7 depicts the onset time of malignancy-related AEs for various DMARDs. Sarilumab demonstrated an earlier median onset time, at 0.58 years (IQR: 0.25–1.25 years). Conversely, the longest was 7.08 years (IQR: 6.17–11.91 years) for hydroxychloroquine. The csDMRADs exhibited a relatively longer median time, ranging from 3.58 to 7.08 years, while the tsDMARDs demonstrated a shorter median time of 0.83–1.67 years.

## 4 Discussion

As the application of DMARDs continues to surge, the associated malignancy AEs have become a matter of great concern. In February 2021, the FDA issued a warning that tofacitinib (Xeljanz) carries a cancer risk compared to TNF inhibitors in older patients. In December 2021, the agency required new and updated warnings for two other JAK inhibitors, Olumiant (baricitinib) and Rinvoq (upadacitinib), due to their shared mechanisms of action with Xeljanz (FDA, 2022b). In fact, a systematic overview of contraindications and special warnings for DMARDs indicated that malignancy risk was mentioned for all drug classes (Skaarup et al., 2024). However, detailed studies are still essential to comprehensively understand the specific cancer risks associated with each DMARD, as this information can enhance medication safety and serve as a critical reference for healthcare practitioners in making informed treatment decisions. Our study offers a comprehensive insight by conducting a retrospective pharmacovigilance analysis based on the FAERS data from the past 5 years. Following data cleansing and deduplication, we identified a total of 17,412 reports pertaining to the potential cancer risk associated with DMARDs. This study represents the first large-scale post-marketing data analysis investigating the correlation between DMARDs and malignancies.

Descriptive analysis in our study has revealed that females have a propensity of 2–3 times to report malignancy AEs compared to males. Patients around 62 years old are more prone to experience such events. The higher prevalence of RA in women compared to men, with a ratio of 2–3 times, and its occurrence at any age, with the peak onset in the sixth decade, may account for this phenomenon (Aletaha and Smolen, 2018). Sound explanations, such as the influence of sex chromosomes and the effects of estrogen on immunity, likely contribute to the female predominance in disease distribution (Cutolo and Straub, 2020). Additionally, tsDMARDs and bDMARDs tend to have a relatively earlier onset of malignancy AEs compared to csDMARDs. This may be because patients receiving these newer biologics are often more closely monitored, which facilitates earlier detection. The shorter time to onset could also be influenced by the fact that these drugs have been in use for a shorter period, limiting our ability to capture longer-term effects within the current study period. Future studies with extended follow-up periods may provide more comprehensive insights into the time to onset of malignancy AEs for these newer compounds.

After consulting with a rheumatologist and combining medical knowledge, we have found that the AEs associated with malignancies for DMARDs generally align with the information provided in the product labeling. However, we have also identified statistically significant signals of unexpected drug AEs that are not listed in the labeling, including methotrexate (myelodysplastic syndrome, neuroendocrine carcinoma of the skin, non-small cell lung cancer, acute myeloid leukaemia and metastases to bone marrow), baricitinib (breast cancer, metastases to liver), upadacitinib (breast cancer female, lung neoplasm malignant, uterine leiomyoma, renal cancer, tongue neoplasm malignant stage unspecified, squamous cell carcinoma), adalimumab (lipoma, acrochordon, neuroma, pituitary tumour, spinal cord neoplasm, pituitary tumour benign, hypergammaglobulinaemia benign monoclonal and rhabdomyosarcoma) and abatacept (squamous cell carcinoma). To enhance understanding of these phenomena, we will undertake an in-depth analysis of the potential pathological mechanisms that underlie the occurrence of malignancy-related AEs across various DMARDs.

### 4.1 csDMARDs and risk of malignancy

In our study, among the csDMARDs, only methotrexate (MTX) exhibited signals of risk associated with malignancies. The oncogenicity of MTX involves the transformation of B cells into premalignant or malignant clones, mediated by EBV and/or cytogenetic effects induced by MTX (L and Sa, 1999). The impaired function of suppressor lymphocytes (Ts)/cytotoxic T lymphocytes (Tc) and natural killer (NK) cells observed in patients with RA leads to deficiencies in EBV-specific immunity, allowing for the growth and proliferation of EBV-infected B lymphocytes. MTX exacerbates Ts/Tc dysfunction by inhibiting polyamines and directly inducing apoptosis, which makes it possible for highly active B cell clones to transform into lymphomas in lymph nodes and synovium (Miyazaki et al., 2007). Additionally, MTX has the potential to induce malignancy in CD5^+^ B cell clones that are commonly expanded in RA. Lastly, it is possible that common genetic or environmental factors that predispose individuals to both RA and lymphoma could be influenced by MTX in a manner that promotes co-carcinogenesis (Zhang et al., 2022).

The aetiological role of MTX in the development of lymphomas is supported by the observation of spontaneous remission of these malignancies in some patients with RA following discontinuation of MTX. Physicians managing patients with RA receiving MTX should remain vigilant for signs and symptoms suggestive of lymphoma, particularly in those with significant comorbidity and severe disease who are more likely to be immunosuppressed. MTX should be ceased if lymphoma appears in these patients, with a period of observation being considered when clinically feasible. Prompt initiation of specific anti-tumor therapy is warranted if there is evidence of organ invasion by proliferating lymphocytes or functional deterioration months later (Wong et al., 2018).

### 4.2 JAKi and risk of malignancy

We observed that, even after adjusting for age, gender, and the effects of concomitant medications, JAKi exhibited the most prevalent and pronounced tumor-related adverse signals at both SMQ and PT levels among the DMARDs included in our analysis. This observation is consistent with prior research indicating that, across 62 randomized controlled trials and 16 long-term extension studies, there was no significant difference in the overall rate of malignancies, including non-melanoma skin cancers, when comparing JAKi to methotrexate (incidence rate ratio (IRR) 0.77; 95% CI 0.35–1.68). However, compared to TNFi, JAKi were linked to a higher occurrence of malignancies (IRR 1.50; 95% CI 1.16–1.94) (Ytterberg et al., 2022). Furthermore, a nationwide cohort study revealed that in real-world clinical settings, the short-term risk of non-melanoma skin cancers was elevated among patients who started treatment with JAKi compared to those using TNFi (Huss et al., 2023).

Regarding the carcinogenic mechanism of JAKi, it is essential to consider the role of JAK. JAK plays a pivotal role in lymphocyte activation, function, and proliferation via its receptor and downstream signal transduction, including tyrosine phosphorylation of the transcription activator signaling. (Owen et al., 2019). The development, maturation, activation, and function of NK cells are tightly controlled by cytokines through the JAK-STAT pathway (Benucci et al., 2022). Research has shown that high tumor expression of NK cell ligands, such as MIC-A/B and ULBP-2, is associated with improved clinical outcomes in various cancers (De Kruijf et al., 2012). Mice lacking NK cells exhibited increased vulnerability to tumors, and in humans, a reduction in NK cell function and infiltration was observed in the tumor microenvironment of high-grade ovarian carcinoma, metastatic lung adenocarcinoma, and gastric cancer. This suggests that a decrease in NK cell-mediated cytotoxicity is associated with an increased risk of cancer incidence and progression (Takanami et al., 2001; Imai et al., 2000). A recent preclinical study demonstrated that JAK inhibition negatively impacts the phenotype and function of NK cells (Meudec et al., 2023). This finding was corroborated by the observation of STAT 5-deficient NK cells exhibiting reduced anti-tumor cytotoxicity in a melanoma mouse model (Gotthardt et al., 2016).

### 4.3 bDMARDs and risk of malignancy

In the initial crude OR analysis, the TNFi adalimumab showed a positive association with an increased risk of malignancies, while the associations for golimumab and infliximab were inconclusive or weak. However, after adjusting for potential confounders, the results suggested that adalimumab may have a protective effect against malignancies, or at the very least, does not increase the risk. In contrast, golimumab and infliximab may be associated with an increased risk of malignancies. It is important to note that, despite multivariate adjustments, there may still be unmeasured confounders to consider. Additionally, the OR value merely reflects an association, not a causal relationship. Therefore, these findings need to be validated in other studies and should be considered in clinical practice alongside other evidence and individual patient circumstances.

The proinflammatory cytokine TNF-α, produced by immune cells including T-cells, B-cells and macrophages, plays a complex role in neoplastic diseases (Wang and Lin, 2008). It exhibits dual functionality: on one side, TNF-α acts as an endogenous tumor promoter by stimulating cancer cell growth, proliferation, invasion, metastasis, and tumor angiogenesis (Devoogdt et al., 2006; Balkwill, 2006). Conversely, it can selectively destroy tumor vasculature, inducing apoptosis and necrosis in cancer cells, and potentially serving as an effector molecule in immune-mediated cancer cell destruction (Kashii et al., 1999). Research has identified two primary mechanisms for its anti-cancer effects. Firstly, TNF-α enhances the effectiveness of liposome-mediated chemotherapy by increasing blood vessel permeability, thereby facilitating drug accumulation at the tumor site. This enhancement of vascular permeability also leads to the accumulation of cytostatic drugs and antibodies within the tumor, aiding in tumor vasculature destruction (Seynhaeve et al., 2007). Secondly, high levels of exogenous TNF-α can directly induce apoptosis in malignant cells, although its cytotoxic effects may only be evident in the presence of other metabolic inhibitors (Balkwill, 2009; Cruceriu et al., 2020). Further research has demonstrated that TNF knockout mice were more susceptible to skin sarcoma induced by 3′-methylcholanthrene (MCA) compared to wild-type mice, indicating that TNF provides protection against MCA-induced sarcoma formation (Swann et al., 2008). Analogous protective effects were also noted in a mouse model with xenografted glioma (Nakagawa et al., 2007).

Following the successful application of TNFi in RA, a variety of other categories of bDMARDs have been developed and are being increasingly utilized. These include rituximab, abatacept, tocilizumab, sarilumab and anakinra (Chen et al., 2024; Patel et al., 2023). Given their recent introduction, there is limited evidence regarding potential links between these treatments and the onset of malignancies. The underlying mechanisms of tumor induction are not fully understood, necessitating further animal studies or case reports to elucidate this in the future.

### 4.4 Limitations

This retrospective analysis has certain inherent constraints. Firstly, the disproportionality analysis employed in pharmacovigilance research poses challenges in establishing causality, necessitating further studies to confirm these findings. Additionally, the FAERS database, being a spontaneous reporting system, has several intrinsic drawbacks, including data redundancies, gaps in information and varied data sources. Moreover, our analysis was limited to FAERS data from 2019 to 2023, potentially excluding recent trends or patterns in AEs that emerged in 2024 or earlier years. This temporal limitation could affect the generalizability and robustness of the identified signals. Despite these limitations, our analysis, based on over eight million records from real-world clinical practice, may provide important clues for guiding future relevant clinical studies. Our research logically and methodically evaluated the potential malignancy risks associated with DMARDs.

## 5 Conclusion

Our study explores the potential associations between DMARDs and AEs linked to malignancy from both the PT and SMQ levels, revealing varying degrees of cancer risks linked to DMARDs, with tsDMARDs exhibiting a notably higher risk. Unreported novel cancer signals, including instances of myelodysplastic syndrome, non-small cell lung cancer and pituitary tumour, have been detected. These findings may play a central role in facilitating the risk-benefit assessment of DMARDs. As additional data emerges, it is essential for regulatory agencies and professional organizations to remain alert and adapt their recommendations accordingly. The ultimate aim must be to deliver patients with the most effective treatments that are both safe and well-tolerated, a goal that necessitates ongoing collaboration and dialogue between the regulatory agencies, the medical community, and pharmaceutical companies.

## Data Availability

The original contributions presented in the study are included in the article/Supplementary Material, further inquiries can be directed to the corresponding author.
