# Supplementary material for: Risks of malignancies related to disease-modifying antirheumatic drugs in rheumatoid arthritis: a pharmacovigilance analysis using the FAERS database
Source: Front Pharmacol. 2024 Nov 13;15:1458500. doi: 10.3389/fphar.2024.1458500 (PMC11598350; doi:10.3389/fphar.2024.1458500)
Supplement: Supplementary file 1 [file DataSheet1.PDF]

Supplementary Material

Table 1. Major algorithms used for pharmacovigilance analysis

Algorithms	Equation	Criteria
ROR	ROR=ad/b/c<br>95%CI=$e^{\ln(ROR)\pm 1.96(1/a+1/b+1/c+1/d)^{\wedge}0.5}$	$ROR_{025} > 1$
IC	IC=$\log_2 a(a+b+c+d)(a+c)(a+b)$<br>95%CI= E(IC) ± 2V(IC)^0.5	$IC_{025} > 0$

Abbreviations: a, number of reports containing both the target drug and target adverse event; b, number of reports containing other adverse event of the target drug; c, number of reports containing the target adverse event of other drugs; d, number of reports containing other drugs and other adverse event. 95%CI, 95% confidence interval; N, the number of reports; E(IC), the IC expectations; V(IC), the variance of IC.

Table 2. Demographic information on malignancy risks with DMARDs.

	Cases, N (%)					
Characteristic	Methotrexate	Leflunomide	Sulfasalazine	Hydroxychloroquine	Upadacitinib	Baricitinib
Total cases	3,132	202	30	83	1,468	323
Gender						
Data available	3,013	180	26	54	1,423	312
Female	2,087	136	14	41	989	218
Male	926	44	12	13	434	94
Age						
Data available	2,536	144	24	49	830	268
<18	0	0	0	0	0	0
18–44	99	14	2	14	28	7
45-64	805	46	4	17	338	87
65-74	917	60	9	16	290	96
>74	715	24	9	2	174	78
Median (IQR)	62.5(47-77.5)	64(52.5-72.75)	73(61.5-79.25)	59(49.5-68.5)	63(47.25-78.25)	63(50.25-75.25)
Reported countries (Top 3)						
1	JP 1143	CA139	CA8	CA40	US645	JP108
2	CA 648	DE22	JP8	US26	CA120	FR34
3	DE 541	FR17	DE3	FR6	JP90	GB30
Outcomes						
Data available	4,655	332	38	144	1,639	393
Hospitalized(HO)	1,189	91	5	45	297	131
Disabled(DS)	120	30	3	18	11	10
Congenital anomaly(CA)	5	2	0	2	0	0
Life threating(LT)	371	21	2	8	52	35
Died(DE)	467	15	2	6	66	37
Other outcomes（OT,RI）	2,503	173	26	65	1,213	180

Table 2. Demographic information on malignancy risks with DMARDs. (continued)

	Cases, N (%)					
Characteristic	Tofacitinib	Adalimumab	Etanercept	Infliximab	Golimumab	Certolizumab Pegol
Total cases	1,900	2,790	1,616	640	764	764
Gender						
Data available	1,851	2,685	1,575	494	701	739
Female	1,363	2,082	1,175	349	525	562
Male	488	603	400	145	176	177
Age						
Data available	1,787	1,487	1,290	402	585	421
<18	1	2	2	0	0	2
18–44	53	108	73	25	35	38
45-64	671	684	520	144	210	173
65-74	646	461	453	137	172	132
>74	416	232	242	96	168	76
Median (IQR)	61.5(46-76.5)	58.5(42-74.5)	59(42.75-74.75)	60.5(46-74.5)	61.5(45-77.5)	59（43.75-73.75）
Reported countries (Top 3)						
1	US996	US1511	US493	CA316	CA190	US260
2	CA305	CA192	CA223	JP89	JP139	CA111
3	JP233	GB161	GB181	BR56	BR86	GB97
Outcomes						
Data available	2,510	3,632	2,065	801	870	1,015
Hospitalized(HO)	581	757	385	151	175	206
Disabled(DS)	23	98	48	18	11	12
Congenital anomaly(CA)	0	23	7	3	0	1
Life threating(LT)	142	107	118	44	35	49
Died(DE)	188	245	138	54	71	76
Other outcomes（OT,RI）	1,576	2,402	1,369	531	578	671

Table 2. Demographic information on malignancy risks with DMARDs. (continued)

	Cases, N (%)				
Characteristic	Anakinra	Tocilizumab	Sarilumab	Abatacept	Rituximab
Total cases	19	872	104	1,693	1,012
Gender					
Data available	19	818	90	1,633	448
Female	12	645	66	1,273	320
Male	7	173	24	360	128
Age					
Data available	9	579	76	1,325	358
<18	0	0	0	3	0
18–44	0	72	9	65	30
45-64	4	243	23	410	143
65-74	1	159	26	466	126
>74	4	105	18	381	59
Median (IQR)	67(55.75-77.75)	62(47.25-76.25)	63.5(50-72.5)	61(43.75-77.75)	60(46.75-72.75)
Reported countries (Top 3)					
1	US10	CA264	JP38	CA530	CA815
2	CA3	JP156	US36	US365	GB45
3	FR3	US140	CA10	JP203	DE44
Outcomes					
Data available	30	1,075	178	2,479	1,340
Hospitalized(HO)	9	316	35	547	329
Disabled(DS)	2	62	8	81	76
Congenital anomaly(CA)	0	6	0	2	2
Life threating(LT)	0	58	14	147	29
Died(DE)	3	65	24	95	66
Other outcomes（OT,RI）	16	568	97	1,607	838

Abbreviations: AT, Austria; CA, Canada; DE, Germany; FR, France; GB, United Kingdom; US, United States; IQR, interquartile range.

Table 3. Signal strength for Preferred Term (PT).

Drugs	PT	Case(n)	ROR(95%CI)	IC(95%CI)
Methotrexate	Lymphoproliferative disorder	332	7.85(6.85-8.99)	2.4(2.2-2.57)
Methotrexate	Diffuse large B-cell lymphoma	208	7.32(6.18-8.67)	2.34(2.07-2.54)
Methotrexate	Epstein Barr virus positive mucocutaneous ulcer	119	17.54(13.33-23.09)	3.01(2.59-3.26)
Methotrexate	Epstein-Barr virus associated lymphoproliferative disorder	98	11.28(8.61-14.77)	2.71(2.27-2.97)
Methotrexate	Angiocentric lymphoma	78	7.99(6.03-10.59)	2.42(1.95-2.72)
Methotrexate	Hodgkin's disease	57	3.04(2.28-4.05)	1.41(0.95-1.78)
Methotrexate	Myelodysplastic syndrome	53	3.49(2.58-4.73)	1.57(1.08-1.95)
Methotrexate	Plasma cell myeloma	39	2.29(1.63-3.22)	1.07(0.54-1.52)
Methotrexate	Neuroendocrine carcinoma of the skin	38	3.39(2.37-4.84)	1.54(0.95-1.97)
Methotrexate	Acute myeloid leukaemia	30	3.48(2.33-5.21)	1.57(0.9-2.04)
Methotrexate	B-cell lymphoma	29	2.21(1.49-3.28)	1.03(0.41-1.54)
Methotrexate	Kaposi's sarcoma	17	2.53(1.51-4.26)	1.19(0.35-1.82)
Methotrexate	T-cell lymphoma	17	5.07(2.9-8.87)	1.98(0.97-2.51)
Methotrexate	Chronic lymphocytic leukaemia	16	2.62(1.53-4.49)	1.23(0.35-1.87)
Methotrexate	Diffuse large B-cell lymphoma stage IV	16	5(2.81-8.89)	1.96(0.92-2.51)
Methotrexate	Extranodal marginal zone B-cell lymphoma (MALT type)	16	5.25(2.94-9.37)	2.01(0.96-2.55)

Methotrexate	Central nervous system lymphoma	15	5.47(2.99-9.98)	2.05(0.95-2.6)
Methotrexate	Metastases to central nervous system	15	3.03(1.73-5.31)	1.4(0.46-2.04)
Methotrexate	Non-small cell lung cancer	15	5.32(2.92-9.69)	2.03(0.93-2.57)
Methotrexate	Peripheral T-cell lymphoma unspecified	15	10.93(5.51-21.69)	2.68(1.36-3.11)
Methotrexate	Epstein-Barr virus associated lymphoma	14	5.4(2.9-10.07)	2.04(0.89-2.6)
Methotrexate	Renal cell carcinoma	13	2.16(1.2-3.88)	1(0.06-1.71)
Methotrexate	Follicular lymphoma	12	4.5(2.33-8.66)	1.85(0.66-2.47)
Methotrexate	Transitional cell carcinoma	11	3.7(1.89-7.22)	1.64(0.45-2.31)
Methotrexate	Metastases to bone marrow	10	6.9(3.21-14.85)	2.28(0.8-2.83)
Methotrexate	Plasmablastic lymphoma	10	131.18(16.79-1024.78)	3.68(1.37-3.78)
Methotrexate	Large granular lymphocytosis	9	2.68(1.31-5.5)	1.26(0.06-2.04)
Methotrexate	Malignant melanoma in situ	9	3.47(1.67-7.24)	1.56(0.27-2.29)
Methotrexate	Metastatic bronchial carcinoma	9	2.68(1.31-5.5)	1.26(0.06-2.04)
Methotrexate	Anaplastic large cell lymphoma T- and null-cell types	8	26.24(7.9-87.13)	3.23(0.98-3.48)
Methotrexate	Histiocytic necrotising lymphadenitis	8	14.99(5.44-41.34)	2.91(0.87-3.28)
Methotrexate	Squamous cell carcinoma of the oral cavity	8	2.76(1.29-5.92)	1.3(0.01-2.11)
Methotrexate	Peripheral T-cell lymphoma	7	91.83(11.3-	3.63(0.89-3.69)

	unspecified stage III		746.37)	
Methotrexate	Anal squamous cell carcinoma	6	3.75(1.51-9.29)	1.65(0.01-2.45)
Methotrexate	Bronchioloalveolar carcinoma	6	5.25(2.04-13.52)	2.01(0.2-2.7)
Methotrexate	Non-small cell lung cancer metastatic	6	3.94(1.58-9.8)	1.7(0.04-2.49)
Methotrexate	Cutaneous B-cell lymphoma	5	10.93(3.34-35.82)	2.68(0.25-3.14)
Methotrexate	Adrenocortical carcinoma	4	17.49(3.91-78.15)	3.01(0.03-3.32)
Methotrexate	Heavy chain disease	4	52.47(5.86-469.48)	3.5(0.06-3.56)
Methotrexate	Metastases to skin	4	17.49(3.91-78.15)	3.01(0.03-3.32)
Upadacitinib	Skin cancer	152	12.58(10.61-14.91)	3.47(3.13-3.63)
Upadacitinib	Neoplasm malignant	142	2.58(2.18-3.05)	1.34(1.08-1.57)
Upadacitinib	Lung neoplasm malignant	77	4.56(3.63-5.74)	2.13(1.73-2.4)
Upadacitinib	Breast cancer	69	2.33(1.83-2.96)	1.2(0.82-1.52)
Upadacitinib	Squamous cell carcinoma of skin	65	25.27(19.17-33.3)	4.31(3.55-4.34)
Upadacitinib	Breast cancer female	52	9.02(6.78-12)	3.05(2.44-3.27)
Upadacitinib	Malignant melanoma	45	4.15(3.08-5.6)	2(1.47-2.34)
Upadacitinib	Prostate cancer	31	2.91(2.04-4.17)	1.51(0.91-1.95)
Upadacitinib	Lymphoma	29	1.71(1.18-2.47)	0.76(0.2-1.26)
Upadacitinib	Squamous cell carcinoma	27	5.05(3.43-7.45)	2.27(1.52-2.65)

Upadacitinib	Colon cancer	21	4.3(2.77-6.66)	2.05(1.23-2.49)
Upadacitinib	Renal cancer	18	7.15(4.42-11.57)	2.74(1.67-3.05)
Upadacitinib	Uterine leiomyoma	18	7.19(4.44-11.62)	2.75(1.68-3.05)
Upadacitinib	Bladder cancer	15	4.07(2.42-6.82)	1.97(0.99-2.47)
Upadacitinib	Plasma cell myeloma	15	5.28(3.14-8.9)	2.33(1.26-2.75)
Upadacitinib	Gastric cancer	14	5.06(2.95-8.67)	2.27(1.17-2.71)
Upadacitinib	Hepatic cancer	13	7.3(4.14-12.85)	2.77(1.46-3.06)
Upadacitinib	Ovarian cancer	13	3.61(2.08-6.29)	1.81(0.78-2.36)
Upadacitinib	Pancreatic carcinoma	13	4.6(2.63-8.03)	2.14(1.03-2.62)
Upadacitinib	Lung neoplasm	12	6.78(3.77-12.2)	2.67(1.33-3)
Upadacitinib	Melanocytic naevus	12	3.96(2.22-7.05)	1.94(0.83-2.47)
Upadacitinib	Leukaemia	11	6.1(3.31-11.24)	2.53(1.18-2.9)
Upadacitinib	Brain neoplasm	10	2.82(1.5-5.29)	1.46(0.36-2.13)
Upadacitinib	Lipoma	9	4.89(2.5-9.58)	2.23(0.83-2.71)
Upadacitinib	Neoplasm	9	2.43(1.25-4.72)	1.26(0.14-1.99)
Upadacitinib	Tongue neoplasm malignant stage unspecified	9	19.1(9.28-39.3)	3.99(1.66-3.66)
Upadacitinib	Bone cancer	8	13.13(6.25-27.63)	3.53(1.37-3.43)
Upadacitinib	Metastases to liver	8	3.25(1.61-6.59)	1.67(0.37-2.33)
Upadacitinib	Renal neoplasm	8	4.12(2.03-8.37)	1.99(0.59-2.56)

Upadacitinib	Oesophageal carcinoma	7	4.45(2.08-9.5)	2.1(0.55-2.64)
Upadacitinib	Benign neoplasm	6	2.82(1.25-6.36)	1.47(0.03-2.25)
Upadacitinib	Bone neoplasm	6	21.75(8.89-53.22)	4.14(1.16-3.58)
Upadacitinib	Invasive ductal breast carcinoma	6	4.14(1.83-9.4)	2(0.36-2.6)
Upadacitinib	Joint neoplasm	6	104.42(31.87-342.16)	5.59(1.25-4)
Upadacitinib	Meningioma	6	6(2.62-13.73)	2.51(0.63-2.89)
Upadacitinib	Rectal cancer	6	4.35(1.92-9.88)	2.07(0.4-2.64)
Upadacitinib	Throat cancer	6	4.42(1.95-10.05)	2.09(0.41-2.65)
Upadacitinib	Metastases to lung	5	3.78(1.55-9.26)	1.87(0.13-2.54)
Upadacitinib	Metastatic neoplasm	5	5.31(2.15-13.09)	2.34(0.37-2.8)
Upadacitinib	Uterine cancer	5	3.6(1.47-8.8)	1.8(0.09-2.5)
Upadacitinib	Benign neoplasm of thyroid gland	4	8.09(2.91-22.55)	2.91(0.34-3.05)
Upadacitinib	Bladder neoplasm	4	4.41(1.61-12.03)	2.08(0.03-2.68)
Upadacitinib	Bowen's disease	4	10.55(3.74-29.77)	3.25(0.43-3.18)
Upadacitinib	Endometrial cancer	4	4.83(1.77-13.23)	2.21(0.08-2.75)
Upadacitinib	Glioblastoma	4	19.34(6.54-57.14)	4(0.56-3.41)
Upadacitinib	Intraductal proliferative breast lesion	4	6.22(2.25-17.14)	2.55(0.22-2.9)
Upadacitinib	Metastases to lymph nodes	4	4.7(1.72-12.86)	2.17(0.07-2.73)

Tofacitinib	Skin cancer	159	2.87(2.43-3.39)	1.39(1.13-1.62)
Tofacitinib	Lung neoplasm malignant	119	1.57(1.31-1.9)	0.61(0.33-0.88)
Tofacitinib	Colon cancer	51	2.44(1.82-3.26)	1.18(0.72-1.57)
Tofacitinib	Breast cancer female	47	1.75(1.3-2.36)	0.75(0.3-1.16)
Tofacitinib	Squamous cell carcinoma	36	1.49(1.06-2.09)	0.54(0.03-1.01)
Tofacitinib	Gastric cancer	29	2.42(1.64-3.56)	1.18(0.55-1.67)
Tofacitinib	Neoplasm	28	1.74(1.18-2.57)	0.75(0.15-1.27)
Tofacitinib	Pancreatic carcinoma	28	2.29(1.55-3.39)	1.1(0.48-1.61)
Tofacitinib	Squamous cell carcinoma of skin	25	1.79(1.19-2.69)	0.78(0.15-1.33)
Tofacitinib	Renal cancer	22	1.93(1.25-2.99)	0.88(0.2-1.46)
Tofacitinib	Leukaemia	17	2.12(1.29-3.51)	1.01(0.21-1.64)
Tofacitinib	Uterine cancer	17	2.94(1.77-4.91)	1.42(0.56-2.01)
Tofacitinib	Lung carcinoma cell type unspecified stage IV	14	7.34(3.96-13.61)	2.48(1.22-2.92)
Tofacitinib	Cervix carcinoma	12	2.49(1.36-4.54)	1.21(0.21-1.91)
Tofacitinib	Neoplasm recurrence	11	9.88(4.77-20.5)	2.77(1.2-3.15)
Tofacitinib	Chronic myeloid leukaemia	10	2.86(1.47-5.56)	1.39(0.24-2.1)
Tofacitinib	Endometrial cancer	10	2.86(1.47-5.56)	1.39(0.24-2.1)
Tofacitinib	Lip and/or oral cavity cancer	8	3.87(1.81-8.28)	1.76(0.35-2.45)
Tofacitinib	Anal cancer	7	4.72(2.06-10.8)	1.99(0.38-2.64)
Tofacitinib	Gastric neoplasm	7	4.72(2.06-10.8)	1.99(0.38-2.64)

Tofacitinib	Bile duct cancer	5	6.74(2.43-18.71)	2.39(0.24-2.92)
Tofacitinib	Ear neoplasm malignant	5	6.74(2.43-18.71)	2.39(0.24-2.92)
Tofacitinib	Lung carcinoma cell type unspecified stage I	4	7.55(2.37-24.07)	2.51(0.02-3)
Tofacitinib	Parathyroid tumour	4	9.43(2.84-31.33)	2.73(0.08-3.11)
Baricitinib	Breast cancer	24	11.09(7.41-16.58)	3.45(2.39-3.55)
Baricitinib	Neoplasm malignant	23	5.68(3.77-8.56)	2.5(1.65-2.83)
Baricitinib	Lung neoplasm malignant	10	7.88(4.23-14.69)	2.97(1.4-3.15)
Baricitinib	Metastases to liver	9	51.17(26.25-99.73)	5.62(2.14-4.01)
Baricitinib	Prostate cancer	8	10.21(5.09-20.48)	3.34(1.36-3.3)
Baricitinib	Diffuse large B-cell lymphoma	6	12.63(5.65-28.24)	3.64(1.14-3.34)
Baricitinib	Lung adenocarcinoma	6	24.29(10.82-54.52)	4.57(1.38-3.59)
Baricitinib	Adenocarcinoma gastric	5	163.54(64.26-416.25)	7.17(1.28-3.79)
Baricitinib	Hepatocellular carcinoma	5	63.7(25.91-156.61)	5.92(1.26-3.68)
Baricitinib	Lung neoplasm	5	37.58(15.43-91.56)	5.19(1.2-3.6)
Baricitinib	Lymphoproliferative disorder	5	6.86(2.85-16.53)	2.77(0.61-2.97)
Baricitinib	Malignant melanoma	5	6.15(2.55-14.81)	2.61(0.54-2.9)
Baricitinib	Thyroid cancer	5	14.69(6.08-	3.86(0.97-3.34)

			35.48)	
Baricitinib	Acute myeloid leukaemia	4	34.82(12.88-94.11)	5.08(0.85-3.47)
Baricitinib	Adenocarcinoma of colon	4	46.99(17.3-127.63)	5.5(0.88-3.52)
Baricitinib	Bronchial carcinoma	4	63.69(23.29-174.11)	5.92(0.9-3.56)
Baricitinib	Cervix carcinoma	4	48.89(17.99-132.88)	5.55(0.88-3.52)
Baricitinib	Diffuse large B-cell lymphoma stage IV	4	89.63(32.45-247.56)	6.38(0.91-3.6)
Baricitinib	Metastases to lung	4	41.73(15.4-113.09)	5.33(0.87-3.5)
Baricitinib	Non-Hodgkin's lymphoma	4	4.03(1.51-10.75)	2.01(0.03-2.62)
Baricitinib	Squamous cell carcinoma	4	9.92(3.71-26.54)	3.3(0.53-3.13)
Baricitinib	Squamous cell carcinoma of skin	4	16.98(6.33-45.57)	4.07(0.71-3.31)
Rituximab	Bladder neoplasm	10	2.67(1.38-5.16)	1.3(0.18-2.03)
Rituximab	Benign neoplasm of thyroid gland	8	3.99(1.87-8.54)	1.8(0.38-2.48)
Rituximab	Lung carcinoma cell type unspecified recurrent	7	12.38(4.8-31.95)	2.99(0.84-3.27)
Rituximab	Gastrointestinal stromal tumour	5	4.23(1.61-11.13)	1.87(0.02-2.61)
Rituximab	Hepatic cancer metastatic	5	4.87(1.83-12.96)	2.03(0.09-2.71)
Infliximab	Gastrointestinal carcinoma	11	2.16(1.17-3.99)	1.05(0.05-1.78)

Golimumab	Neoplasm	20	2.09(1.33-3.28)	1.02(0.29-1.59)
Golimumab	Cervix carcinoma	8	2.73(1.33-5.63)	1.38(0.13-2.13)
Golimumab	Clear cell renal cell carcinoma	4	7.64(2.57-22.71)	2.67(0.16-3.02)
Etanercept	Colon cancer	50	2.18(1.62-2.92)	1.03(0.57-1.43)
Etanercept	Neoplasm	33	1.91(1.33-2.73)	0.86(0.3-1.34)
Etanercept	Leukaemia	21	2.46(1.56-3.89)	1.19(0.45-1.75)
Etanercept	Bone cancer	9	2.98(1.47-6.05)	1.43(0.2-2.17)
Etanercept	Lymphocytic leukaemia	6	5.44(2.17-13.63)	2.13(0.3-2.76)
Etanercept	Colorectal adenocarcinoma	4	34.47(6.31-188.2)	3.6(0.16-3.54)
Certolizumab_Pegol	Leukaemia	14	2.76(1.59-4.76)	1.38(0.45-2.01)
Certolizumab_Pegol	Gastrointestinal neoplasm	4	7.58(2.53-22.67)	2.65(0.15-3.02)
Adalimumab	Skin cancer	134	1.71(1.43-2.05)	0.71(0.44-0.96)
Adalimumab	Breast cancer female	88	2.58(2.06-3.24)	1.22(0.87-1.53)
Adalimumab	Neoplasm	85	4.72(3.69-6.04)	1.91(1.51-2.21)
Adalimumab	Uterine leiomyoma	65	5.2(3.9-6.91)	2.01(1.54-2.35)
Adalimumab	Brain neoplasm	64	3.43(2.61-4.51)	1.56(1.12-1.9)
Adalimumab	Melanocytic naevus	57	3.56(2.66-4.76)	1.6(1.13-1.96)
Adalimumab	Lipoma	47	5.27(3.76-7.37)	2.03(1.46-2.4)
Adalimumab	Bladder cancer	34	1.54(1.08-2.19)	0.57(0.03-1.06)

Adalimumab	Lung neoplasm	30	3.01(2.03-4.48)	1.41(0.76-1.89)
Adalimumab	Leukaemia	27	2.62(1.73-3.95)	1.24(0.57-1.75)
Adalimumab	Renal cancer	26	1.68(1.12-2.53)	0.69(0.06-1.23)
Adalimumab	Benign breast neoplasm	23	6.83(4.14-11.27)	2.29(1.37-2.74)
Adalimumab	Hepatic cancer	22	2.06(1.32-3.22)	0.94(0.24-1.52)
Adalimumab	Bladder neoplasm	19	4.06(2.43-6.77)	1.75(0.85-2.29)
Adalimumab	Uterine cancer	19	2.43(1.49-3.95)	1.15(0.36-1.74)
Adalimumab	Brain neoplasm malignant	12	7.13(3.55-14.33)	2.33(0.98-2.85)
Adalimumab	Bone cancer	11	3.01(1.57-5.78)	1.4(0.29-2.11)
Adalimumab	Endometrial cancer	11	2.31(1.22-4.38)	1.09(0.05-1.84)
Adalimumab	Hepatic neoplasm	11	3.5(1.8-6.78)	1.58(0.42-2.26)
Adalimumab	Intraductal proliferative breast lesion	11	3.07(1.6-5.9)	1.43(0.31-2.13)
Adalimumab	Acrochordon	10	5.69(2.72-11.91)	2.11(0.7-2.7)
Adalimumab	Leiomyoma	9	5.86(2.68-12.79)	2.14(0.63-2.73)
Adalimumab	Bone neoplasm	8	4.97(2.21-11.16)	1.97(0.44-2.62)
Adalimumab	Colon neoplasm	8	6.83(2.92-15.97)	2.29(0.61-2.86)
Adalimumab	Hypergammaglobulinaemia benign monoclonal	8	3.31(1.53-7.17)	1.52(0.16-2.28)
Adalimumab	Neuroma	8	7.29(3.09-17.19)	2.35(0.64-2.9)
Adalimumab	Pituitary tumour	8	5.21(2.31-11.75)	2.02(0.47-2.66)

Adalimumab	Pituitary tumour benign	8	3.53(1.62-7.67)	1.59(0.21-2.34)
Adalimumab	Abdominal neoplasm	7	9.57(3.64-25.13)	2.59(0.62-3.07)
Adalimumab	Ear neoplasm	7	7.36(2.94-18.45)	2.36(0.52-2.92)
Adalimumab	Gastric neoplasm	7	3.42(1.49-7.82)	1.55(0.08-2.34)
Adalimumab	Salivary gland neoplasm	7	7.36(2.94-18.45)	2.36(0.52-2.92)
Adalimumab	Benign lymph node neoplasm	6	16.4(5.01-53.74)	3(0.57-3.32)
Adalimumab	Gastrointestinal neoplasm	6	5.86(2.25-15.24)	2.14(0.27-2.78)
Adalimumab	Nasal neoplasm	6	6.31(2.4-16.6)	2.21(0.3-2.83)
Adalimumab	Ocular neoplasm	6	5.13(2.01-13.1)	2(0.2-2.69)
Adalimumab	Spinal cord neoplasm	6	6.83(2.56-18.21)	2.29(0.33-2.88)
Adalimumab	Adenoma benign	5	5.69(2.01-16.16)	2.11(0.07-2.79)
Adalimumab	Haemangioma of liver	5	7.59(2.54-22.66)	2.39(0.17-2.96)
Adalimumab	Nasal cavity cancer	5	5.26(1.87-14.74)	2.03(0.03-2.74)
Adalimumab	Tumour rupture	5	22.78(5.44-95.32)	3.2(0.37-3.42)
Adalimumab	Rhabdomyosarcoma	4	54.67(6.11-489.13)	3.55(0.08-3.57)
Abatacept	Skin cancer	107	1.89(1.55-2.31)	0.86(0.56-1.14)
Abatacept	Lung neoplasm malignant	95	1.27(1.03-1.57)	0.33(0.02-0.63)
Abatacept	Squamous cell carcinoma	45	1.96(1.44-2.66)	0.9(0.43-1.32)
Abatacept	Neoplasm	29	1.87(1.27-2.73)	0.84(0.25-1.35)
Abatacept	Squamous cell carcinoma of skin	26	1.92(1.28-2.87)	0.88(0.25-1.41)

Abatacept	Vaginal cancer	4	9.71(2.92-32.25)	2.77(0.09-3.13)

Note: Only significant signals are recorded. Leflunomide, sulfasalazine, hydroxychloroquine, anakinra, tocilizumab, and sarilumab lack significant signals, and iguratimod, filgotinib, and peficitinib lack relevant reports, and are therefore not included in the table.

Table 4. Signal strength for SMQ.

Drugs	SMQ	Case(n)	ROR(95%CI)	IC(95%CI)
Methotrexate	Malignant lymphomas	1089	2.81(2.63-3)	1.3(1.21-1.4)
Tofacitinib	Uterine and fallopian tube neoplasms, malignant and unspecified	155	1.3(1.1-1.53)	0.36(0.12-0.59)
Upadacitinib	Malignancies	2171	3.16(3.03-3.31)	1.6(1.53-1.66)
Upadacitinib	Skin neoplasms, malignant and unspecified	429	3.56(3.23-3.92)	1.79(1.64-1.92)
Upadacitinib	Tumour lysis syndrome	313	1.52(1.36-1.7)	0.59(0.43-0.75)
Upadacitinib	Uterine and fallopian tube neoplasms, malignant and unspecified	276	12.63(11.14-14.32)	3.5(3.26-3.63)
Upadacitinib	Prostate neoplasms, malignant and unspecified	38	3.65(2.64-5.04)	1.83(1.27-2.21)
Upadacitinib	Ovarian neoplasms, malignant and unspecified	32	4.06(2.85-5.79)	1.98(1.34-2.37)
Adalimumab	Uterine and fallopian tube neoplasms, malignant and unspecified	561	4.04(3.68-4.44)	1.75(1.61-1.88)
Baricitinib	Malignancies	416	5.9(5.34-6.52)	2.47(2.31-2.6)
Baricitinib	Breast neoplasms, malignant and	62	3.01(2.34-3.86)	1.58(1.17-1.9)

	unspecified			
Baricitinib	Tumour lysis syndrome	61	2.84(2.2-3.65)	1.49(1.08-1.82)
Baricitinib	Malignant lymphomas	32	5.41(3.82-7.66)	2.42(1.74-2.75)
Baricitinib	Skin neoplasms, malignant and unspecified	29	2.24(1.56-3.23)	1.16(0.57-1.63)
Baricitinib	Uterine and fallopian tube neoplasms, malignant and unspecified	13	5.13(2.97-8.85)	2.35(1.21-2.75)
Baricitinib	Prostate neoplasms, malignant and unspecified	9	8.13(4.22-15.68)	3.01(1.32-3.16)
Baricitinib	Ovarian neoplasms, malignant and unspecified	4	4.74(1.78-12.67)	2.24(0.14-2.73)
Golimumab	Uterine and fallopian tube neoplasms, malignant and unspecified	150	2.03(1.72-2.39)	0.97(0.72-1.21)

Note: Only significant signals are recorded. Leflunomide, sulfasalazine, hydroxychloroquine, anakinra, tocilizumab, abatacept, etanercept, rituximab, certolizumab_pegol, infliximab and sarilumab lack significant signals, and are therefore not included in the table.

Table 5. Top 10 concomitant drugs with DMARDs

Concomitant drugs	N (%)	AEs (FDA label information)
methotrexate	17,372 (17.60%)	malignant lymphomas, tumor lysis syndrome
tofacitinib citrate	14,344 (14.53%)	malignancy and lymphoproliferative disorders
prednisone	9,623 (9.75%)	not common
hydroxychloroquine	5,898 (5.97%)	not common

leflunomide	5,891 (5.97%)	malignancy and lymphoproliferative disorders
sulfasalazine	4,968 (5.03%)	carcinogenesis: Two-year oral carcinogenicity studies were conducted in male and female F344/N rats and B6C3F1 mice. Sulfasalazine was tested at 84 (496 mg/m^2), 168 (991 mg/m^2), and337.5 (1,991 mg/m2) mg/kg/day doses in rats. A statistically significant increase in the female rats, two (4%) of the 337.5 mg/kg rats had transitional cell papilloma of the kidney. The increased incidence of neoplasms in the urinary bladder and kidney of rats was also associated with an increase in the renal calculi formation and hyperplasia of transitional cell epithelium. For the mouse study, sulfasalazine was tested at 675 (2025 mg/m^2), 1,350 (4,050 mg/m^2), and 2,700 (8,100 mg/m^2) mg/kg/day. The incidence of hepatocellular adenoma or carcinoma in male and female mice was significantly greater than the control at all doses tested.
folic acid	3,666 (3.71%)	Not common
prednisolone	2,913 (2.95%)	Not common
celecoxib	2,201 (2.23%)	Not common
methylprednisolone acetate	1,997 (2.02%)	Not common
